# Seasonal influence on cognitive and psycho-physiological responses to a single 11-h day of work in outdoor mine industry workers

**DOI:** 10.1080/23328940.2023.2208516

**Published:** 2023-05-12

**Authors:** Sarah M. Taggart, Olivier Girard, Grant J. Landers, Ullrich K. H. Ecker, Karen E. Wallman

**Affiliations:** a School of Human Sciences (Sport Science, Exercise and Health), The University of Western Australia, Crawley, WA, Australia; b School of Psychological Science, The University of Western Australia, Crawley, WA, Australia

**Keywords:** Heat stress, thermal strain, cognition, core temperature, occupational settings, dehydration

## Abstract

This study investigated the seasonal effects that working outdoors had on various parameters in mining industry workers over the course of a work-shift. Workers (*n* = 27) were assessed in summer (33.3 ± 4.2°C, 38 ± 18% RH; *n* = 13, age = 46 ± 14 y, BMI = 29.1 ± 5.7 kg/m^2^) and winter (23.6 ± 5.1°C, 39 ± 20% RH; *n* = 14, age = 44 ± 12 y, BMI = 31.2 ± 4.1 kg/m^2^). Core temperature and heart-rate were measured continuously (analyzed at five time points), while perceptual measures, cognitive and manual dexterity performance were assessed at various times over an 11-h shift at the start of a 14-day swing. Hydration was assessed (urine specific gravity) pre- and post-shift. Working memory was impaired in summer compared to winter (−10%; *p* = 0.039), however did not change throughout the shift. Processing efficiency was significantly reduced at 12 pm (−12%; *p* = 0.005) and 5 pm (−21%; *p* < 0.001) compared to 9 am, irrespective of season (*p* > 0.05). Manual dexterity (dominant-hand) improved over the shift (+13%, *p* = 0.002), but was not different between seasons. Perceived fatigue had no main effect of season or shift. Core temperature, heart-rate, thermal sensation and rating of perceived exertion increased throughout the shift, with only core temperature and thermal sensation showing a seasonal effect (summer: +0.33°C, +18%, respectively; *p* < 0.002). Notably, 23% of workers in summer and 64% in winter started work significantly dehydrated, with 54% and 64% in summer and winter, respectively, finishing work with significant to serious dehydration. Impairment in working memory in summer combined with high levels of dehydration over the work-shift reinforces the need for workplace education on the importance of hydration and risk of occupation heat stress.

**Abbreviations:** Core temperature: T_c_; Fly-in fly-out: FIFO; Ratings of perceived exertion: RPE; Relative humidity: RH; Urinary specific gravity: USG; Wet bulb globe temperature: WBGT.

## Introduction

Working outdoors during the summer months is a major challenge for many occupations [1]. For instance, ambient temperatures on mine sites during summer in Australia often exceed 30°C wet bulb globe temperature (WBGT) [2]. This, combined with the wearing of required personal protective equipment, can result in a rising core temperature (T_c_), cardiovascular strain, and in some cases, significant dehydration over the course of the working day (also known as a shift [3]). Excessive thermal strain can lead to physical and cognitive decrements, which in turn can elevate the risk of heat-related illnesses and hence workplace accidents [4,5].

To date, only a few studies have assessed the consequences of working in extreme heat in field settings on cognitive function. For instance, Mazloumi et al. [6] assessed workers in the steel and iron casting industry during the morning (9.00 am−12.00 pm) of a shift, whilst working in hot (32.9°C WBGT) and cool (16.7°C WBGT) conditions. A greater number of errors and extended reaction time on cognitive tasks (Stroop test: neutral, congruent and incongruent trials) were reported in the hotter condition. However, as perceptual and physiological responses were not assessed by Mazloumi et al. [6], it cannot be ascertained if deteriorated cognitive functioning in the heat was primarily due to higher-than-normal dehydration, physiological strain, and/or thermal discomfort. Conversely, Girard et al. [7] reported that cognitive ability (recognition memory, executive function and working memory) was unaffected by seasonal heat stress (Summer: ~41°C; Winter: ~17°C) in oil-and-gas workers in the Middle-East. However, these authors acknowledged that all testing occurred in a temperature-controlled room (22–24°C) and that participants were both living and working in warm conditions for at least 3–4 months prior to testing, which may have reduced the impact of heat stress on these responses.

Solar radiation is another factor to be considered when working outdoors as prolonged sun exposure can contribute to increases in T_c_ and dehydration, in turn contributing to thermal discomfort and impaired physical work capacity [8]. For instance, 109 construction and agricultural workers performing manual labor in the sun was associated with workers being four times more likely to experience dizziness and twice as likely to suffer heat strain symptoms compared to performing the same work in the shade [9]. By studying the effect of simulated solar radiation on motor-cognitive performance, Piil et al. [10] found that prolonged exposure to heat generated from simulated solar radiation (in a lab) impaired performance on simple and complex motor tasks, and combined-motor cognitive tasks. There is a need for more in-field research in occupational settings on the effect of solar radiation on cognitive performance.

Cognitive function can also be impaired by mental and physical fatigue as a result of demanding manual labor and exertion [11,12]. Indeed, fatigue-related incidents represent one of the greatest risk factors in industrial settings, with ~80% of industrial incidents likely due to human errors [13,14]. Reportedly, the relative risk of workplace incidents occurring during 10-h and 12-h shifts compared to an 8-h shift increased by +13% and +27%, respectively, regardless of environmental temperature [15]. Workplace fatigue, and its negative consequences on cognitive [10] and physical performance [16], can be detrimental to workers’ health and safety, as well as to their productivity [17], if not monitored and carefully managed.

Fly-in-fly-out (FIFO) workers in the North-West of Australia typically spend one to four weeks on site at a time (described as a “swing”), interspersed with one to two weeks at home in between each swing. Little is known about cognitive and psycho-physiological responses to a typical shift in FIFO mine service workers when traveling from their residential city where ambient conditions are typically cooler than their workplace. Importantly, Donoghue et al. [18] reported that more cases of heat exhaustion occur in miners during the first shift of their swing. One potential reason may relate to inadequate fluid intake (i.e. state of dehydration) as evidenced by raised serum osmolality, urea creatinine, and urinary specific gravity (USG) [18]. However, Donoghue et al. [18] did not assess cognitive function, perceived fatigue, thermal discomfort, or physiological variables, rather only determined blood measures and USG once a worker experienced symptoms of heat exhaustion. Further, Piil et al. [19] reported that 70% of 139 workers across five industries in Europe commenced work with a USG≥1.020 (significant to serious dehydration). However, these authors also did not assess the impact that dehydration may have had on cognitive performance in the field.

Previous research has been restricted to pre- *versus* post-shift comparisons and only assessed workers on one working day, not necessarily at the beginning of their swing [6,7]. Little is known about the time-course adjustments in cognitive, manual dexterity and psycho-physiological variables over the duration of a work shift at the beginning of a swing in the heat.

Therefore, this study investigated cognitive, manual dexterity and psycho-physiological responses in outdoor FIFO mining workers at various time-points over the course of a work shift at the start of their swing during summer compared to winter months. It was hypothesized that cognitive and manual dexterity function would deteriorate over the course of a shift, accompanied by progressive increases in perceptual and physiological responses, with these changes being more pronounced in hotter conditions (summer) compared to temperate conditions (winter).

## Methods

### Participants

A repeated measures ANOVA power calculation (a = 0.05, 1-β = 0.95) was conducted with G*Power (Version 3.1.9.3) to determine a sample size based on our primary variable: cognitive performance. Based on the existing literature, the average effect size for a difference in cognitive performance, derived from a random movement generated working memory task is *1.6* [20]. To express our results with 95% confidence, a minimum sample of 12 participants per group was calculated for this study. Therefore, a cohort of 27 male workers (summer: n = 13 [grounds staff = 3, electricians = 2, plumbers = 2, carpenters = 2, refrigeration technicians = 4]; winter: n = 14 [grounds staff = 4, electricians = 2, plumbers = 2, carpenters = 3, refrigeration technicians = 3]) volunteered for this study. Physical characteristics are described in Table 1. Participants were FIFO employees working a 14-day on, 7-day at home roster on a mine site village in the Pilbara region, in the North of Western Australia. All participants resided in Perth (and surrounding areas), Western Australia when on their week at home. Participants were informed of details and requirements of the study before providing informed consent. Ethics approval was granted by the Human Research Ethics Office of the University of Western Australia (RA/4/20/4537) [21].

**Table 1.** Demographic and anthropometric characteristics of participants recruited for the Summer and Winter seasons.

	Age (y)	Height (m)	Body mass (kg)	BMI (kg/m^2^)	Waist to hip	Length of employment (y)
Summer *n=13*	46±14	1.75±0.06	88.9±18.3	29.1±5.7	0.94±0.08	2.1±1.9
Winter *n=14*	44±12	1.77±0.08	98.4±18.5	31.2±4.1	0.97±0.07	1.6±1.7

*Note that no significant differences (p>0.05) were found between summer and winter* populations for any variable assessed.

### Study design

Participants were assessed in respect to cognitive, manual dexterity and psycho-physiological variables over the course of a shift at the start of a swing in hot (March in late summer; average WBGT during the day: ~29.6ºC WBGT [range: 18.8–35.4°C WBGT) and temperate (July in winter; average WBGT during the day: ~20.2°C WBGT [range: 7.2–25.8°C WBGT]) environmental conditions. Three out of 27 participants were tested during both summer and winter months due to workplace attrition rates. Participants underwent a familiarization session on the first shift (day 1) of their 14-day swing after recruitment. They were then tested over the course of a work shift on either day 2 or 3 of this swing. During their 11-h shift, T_c_, heart-rate, thermal sensation, thermal comfort and fatigue, were tested in participants at the start (6–7 am), mid-morning (9–10 am), mid-point (12–1 pm), mid-afternoon (2–3 pm) and end of their daily shift (5–6 pm). Cognitive function and manual dexterity were assessed at the start, mid-point and end of each daily shift. Participants wore the same clothing for each testing session (steel cap boots, yellow-high visibility long sleeve shirt, trousers, and a hat). Food and fluid consumption for the 11-h shift were recorded in diaries, as to determine total fluid intake (i.e. water, coffee, tea, soft drink).

### Familiarization session

Following recruitment, anthropometric measurements, including waist-to-hip ratio and body-mass index were determined (Table 1). Demographic information regarding age, home address, length of employment, occupation, smoking status, and ethnicity were collected. Participants were introduced to all the physiological equipment, and perceptual scales were carefully explained. They also performed the manual dexterity and cognitive tasks (counting span task) five times each in order to reduce any potential learning effect [14].

### Protocol

Upon arrival to work, participants were fitted with a heart-rate monitor and an Actigraph. Participants provided a urine sample during the 30-min period prior to the start of their shift. Next, they attended a ~25 min pre-work meeting where they were assigned their tasks for the day. After this meeting, participants attended baseline testing (described below), which was conducted outdoors in a seated position. The baseline test battery was replicated at mid-shift and end-of-shift time points. Throughout the workday participants conducted a broad range of tasks such as digging, carrying light to heavy loads, driving vehicles, walking, working with tools, gardening and installation of utilities. Workers conducted majority of their tasks (~80%) outdoors throughout their shift.

### Testing procedures

Numerous testing stations were set up, and participants rotated through them in order to assess: (1) manual dexterity and cognitive performance, (2) fatigue, and (3) heart-rate, T_c_, and perceptual measures including thermal sensation, thermal comfort, and rating of perceived exertion. Tests were performed in an invariant order, for a given participant in all their testing sessions. Environmental conditions (ambient temperature, globe temperature, WBGT, and relative humidity) were monitored hourly via the QuesTEMP 32 (TSI Incorporated, USA; accuracy±0.5°C), while wind speed was measured at similar intervals via a digital anemometer (Model: AM-4203 HA, Lutron Electronic Enterprise Co., LTD., Taiwan; accuracy±0.1 km/h).

### Physiological responses

Core temperature was recorded via an ingestible radio-telemetric thermistor using a CorTemp Data Recorder 262K device (CorTemp HQ Inc., Palmetto, USA; accuracy±0.1°C). Capsules were provided to the workers the day before with instructions to swallow it immediately before sleep time. Heart-rate was measured continuously throughout the work-shift via a chest-based monitor (Polar H7, Finland). Participants were fitted with accelerometers (Actigraph GT3×, Pensacola, USA) located on the hip (left side of their belt). Data was recorded continuously (epoch 30 Hz) during the work-shift and was downloaded using ActiLife (Actilife, version 6.13.4, Pensacola, USA), with the total number of steps taken over the duration of the shift determined. Participants provided a urine sample for the measurement of USG using a hand-held refractometer (ATAGO Model URC-N_E_, Japan). Values for USG were defined as *“well hydrated”* < 1.010, *“minimal dehydration”* 1.010–1.020, *“significant dehydration”* 1.021–1.030 and “*serious dehydrated”* > 1.030 [22].

### Perceptual responses

Thermal sensation (0 [very cold] to 20 [very hot]) and thermal comfort (0 [very comfortable] to 20 [very uncomfortable]) were recorded using visual analogue scales ranging from green to red and white to black, respectively [10]. The corresponding scores were only visible to the researcher, with higher thermal sensation and thermal comfort scores representing *feeling hotter* and *less comfortable*, respectively. Ratings of perceived exertion (RPE; 6 [no exertion at all] to 20 [maximal exertion]) were measured using the Borg scale (16, [23]. The Multidimensional Fatigue Scale was used to quantify physical, mental and general fatigue as well as motivation and activity, and this scale has been previously validated in army recruits and junior doctors [17]. Briefly, responses are scored on a scale of 1 (Yes, this is true) to 5 (No, this is not true), with higher scores representing greater levels of fatigue, with each sub-dimension including four questions each.

### Manual dexterity and cognitive function

The Purdue pegboard task (Model 32020, J.A Preston Corporation, New York) was used to assess manual dexterity performance (i.e. concentration, fine motor skills, and hand-eye coordination). Participants had to place as many pegs as possible in a row of holes (one at a time) in 30 s, one hand at a time. Test-retest reliability assessment of the Purdue Pegboard task has been reported by Palejwala et al. [18] with typical error scores and coefficients of variation of±0.5 and 3.1% for the dominant hand and of±0.7 and 4.4% for the non-dominant hand. Working memory capacity and processing efficiency was assessed using a modified version of the Counting Span task (Inquisit Lab 6, Millisecond Software, Seattle, USA) which takes~5 min to complete [19]. This task requires participants to count the number of green dots on cards containing yellow and green dots. After a set number of cards, participants then have to recall the sequence of count numbers in the order of the presented cards. Set size (i.e. the number of cards) thereby increases from 2 to 7 across task trials. The test terminates when two consecutive sequences are recalled incorrectly. Counting latency (time taken to count the green dots), first recall latency (time taken to recall the first number in the sequence), subsequent recall latency (time taken to recall each subsequent number), recall latency (average time taken to recall all numbers in a trial), number of cards counted correctly (correct counting responses), number of counts recalled correctly (correct recall responses) and counting span were recorded. Individual counting and recall latency times (ms) were aggregated across all trials with the same number of green dots, or within the relevant serial position, respectively. There were fewer responses for later recall positions – especially serial positions 6 and 7 – because there were fewer trials with these positions (whereas every trial had positions 1 and 2 and many had positions 3–5). Data from these positions need to be interpreted with caution. To reduce any possible learning effects, different number sequences were randomly assigned such that participants never received the same sequence of numbers (i.e. dot counts) throughout their testing sessions.

### Statistical analysis

Data are expressed as mean ± standard deviation. Statistical analysis was conducted using R studio 1.4.1717. Linear mixed model analysis (obtained using the *lmer* function) was used to compare all dependent variables, with season (summer and winter) and shift (6 am, 9 am, 12 pm, 3 pm and 5 pm) included as fixed effects and random intercepts for participants. P-values were extracted from these mixed models using the *Anova* function in R studio. Where appropriate, post hoc comparisons using *Tukey LSD* were conducted. Statistical significance was accepted at *p* < 0.05. Cohen’s *d* effect sizes with ±95% confidence intervals were calculated for primary variables (working memory, processing efficiency manual dexterity and fatigue) between and within seasons, with only moderate (*0.50–0.79*) to large (*>0.80*) effect sizes reported. A Pearson’s correlation was conducted to determine associations of USG with counting span, correct counting responses and correct recall responses for summer and winter separately. The correlation coefficient was interpreted as either negligible (0–0.10), weak (0.10–0.39), moderate (0.40–0.69), strong (0.70–0.89) or very strong (0.90–1.00).

## Results

Mean ambient temperature (33.3 ± 4.2 *vs*. 23.6 ± 5.1°C; *p* < 0001), globe temperature (42.0 ± 3.1 vs. 30.9 ± 4.4°C), and WBGT (29.6 ± 2.8 *vs*. 20.2 ± 4.3°C; *p* < 0.001), but not relative humidity (38 ± 18 *vs*. 39 ± 20%; *p* = 0.907) and wind speed (8.0 ± 7.9 *vs*. 6.7 ± 4.2 km/h; *p* = 0.108), were significantly greater in summer (*n* = 6) compared to winter (*n* = 5). Maximum average temperature was recorded at 12 pm in summer (37.1 ± 2.4°C) and 3 pm in winter (27.7 ± 3.1°C), with minimum average temperature recorded at 6 am in both summer (25.8 ± 2.9°C) and winter (15.0 ± 4.9°C).

### Cognitive performance

#### Processing efficiency

##### Counting latency

**Figure 1.**
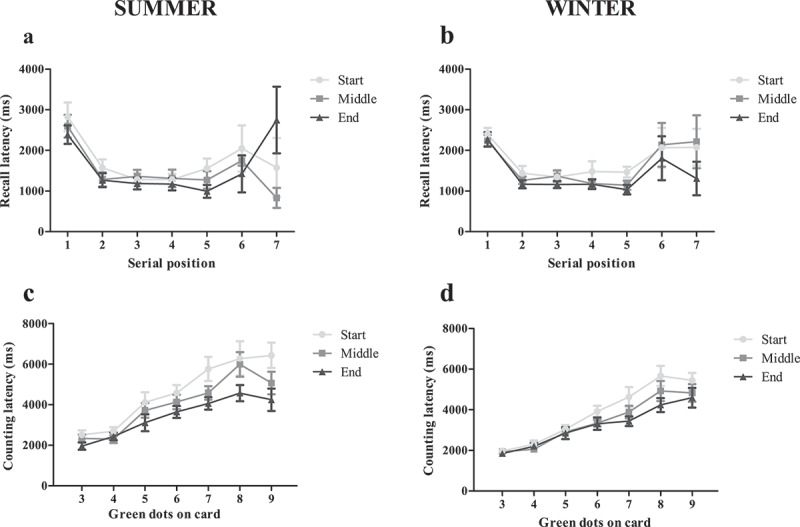
Mean response latencies in the counting span task over the duration of a shift (start (6 am), middle (12 pm) and end (5 pm)). Recall latencies across serial positions (A & B) and counting latencies across number of green dots (C & D) in summer (*n*=13) and winter (*n*=14) over the course of a shift. Recall latency did not significantly differ (*p*>0.05) between seasons or over the course of a shift, however first recall latency (serial position 1) was significantly greater than subsequent recall latency (serial positions 2–7; note that data from positions 6 and 7 are noisy). Irrespective of season, mean counting latency was significantly longer at the start of a shift compared to the middle and end of a shift (*p*<0.05).

There was no significant main effect of season in that counting latency scores for summer (4025 ± 2015 ms) did not differ significantly from scores in winter (3492 ± 1630 ms; p = 0.123; Figure 1). There was a main effect of shift on counting latency (p < 0.001), with mean latency being significantly longer at 6 am (4224 ± 2141 ms) compared to 12 pm (3704 ± 1742 ms; p = 0.005) and 5 pm (3317 ± 1482 ms; p < 0.001). There was no significant interaction effect for counting latency (p = 0.240). There was a moderate effect size for counting latency where in summer, there was a tendency for it to be longer at 6 am compared to 5 pm (*d = 0.60*, [−0.23, 1.33]).

##### Correct counting responses

Correct counting responses had a significant main effect for season (p = 0.022), where more correct responses were provided in winter (49 ± 9) as opposed to summer (43 ± 10; *d = 0.62–0.83* [−0.20, 1.55]; Table 2). There was no significant main effect of shift (p = 0.530). No interaction between season and shift was present for counting correct responses (p = 0.384). There was a weak correlation between USG values (decreased) and correct counting responses (increased) in summer (r = −0.33), whereas both of these variables increased in winter resulting in a weak correlation (r = 0.11).

**Table 2.** Processing speed, working memory and manual dexterity results at the start (6 am), middle (12 pm) and end (5 pm) of shift. Counting correct responses, recall correct responses, counting span score out of 7, and manual dexterity performance for dominant and non-dominant hand (out of 25).

	Summer (*n*=13)			Winter (*n*=14)			ANOVA *P* - Value		
	6 am	12 pm	5 pm	6 am	12 pm	5 pm	Season	Shift	Interaction
**Processing speed**									
Correct counting responses	45±9	41±12	44±10	50±7	50±7	47±12	0.022	0.531	0.384
**Working memory**									
Correct recall responses	38±11	34±12	39±10	43±10	51±8	40±13	0.354	0.120	0.268
Counting span	5.09 ±1.65	4.57 ±1.40	5.32 ±0.87	5.30 ±1.45	5.71 ±0.93	5.41 ±1.40	0.039	0.752	0.167
**Manual Dexterity**									
Dominant hand	15±2	16±2	16±3	15±2	16±2	17±2	0.735	0.002	0.618
Non-dominant hand	14±3	15±3	15±3	15±2	16±2	15±2	0.343	0.030	0.205

#### Working memory

##### Recall latency

There were no significant main effects of shift (*p* = 0.181) or season (*p* = 0.425) on recall latency, nor were there any significant interaction effects between season and shift for recall latency (*p* = 0.972; Figure 1). First recall latency (2446 ± 843 ms) was significantly longer than subsequent response latencies (1458 ± 1124 ms; *p* < 0.001), irrespective of season.

##### Correct recall responses

There were no main effects for correct recall responses on shift or season (Table 2). There was no interaction between swing and shift for correct recall responses. There was a moderate effect size for correct recall responses, where at 12 pm in winter there was a tendency for more correct responses to be recalled compared to 12 pm in summer (*d = 0.69* [−0.14, 1.41). There was a moderate negative correlation between USG values (decreased) and correct recall responses (increased/improved) in summer (*r* = −0.50), whereas both these variables increased in winter (*r* = 0.27) resulting in a weak correlation.

##### Counting span

There was a significant main effect of season (*p* = 0.039) on counting span scores, where scores were significantly lower in summer (4.99 ± 1.35) compared to winter (5.55 ± 1.25). Counting span did not significantly differ throughout the shift (*p* = 0.752; Table 2). There was no significant interaction effect between season and shift (*p* = 0.167). Effect sizes were found in summer between 12 pm and 5 pm (d = −0.64 [−1.38, 0.19]), and between summer and winter for 12 pm scores (d = −0.97 [−1.68, −0.10]). A weak correlation was found between USG values (decreased) and counting span scores (increased) in summer (*r* = −0.34). In winter, there was a weak correlation between these variables, where both increased (*r* = 0.17).

### Manual dexterity

There were significant main effects of shift for the dominant and non-dominant hand (*p* = 0.002 and *p* = 0.030, respectively) but no effect of season (*p* = 0.735 and *p* = 0.343, respectively). For the non-dominant hand, performance was overall significantly better at 12 pm (15 ± 2) compared to 6 am (14 ± 2; *p* = 0.025). Performance with the dominant hand (17 ± 3) was significantly better at 5 pm compared to 6 am (15 ± 2; *p* < 0.05). There were no significant interaction effects for either the dominant (*p* = 0.618) or non-dominant (*p* = 0.205) hand (Table 2). For the dominant hand, effect sizes were found in summer at 6 am to 12 pm and 5 pm (*d=−0.50* [−1.24, 0.32]) and in winter at 6 am to 12 pm and 5 pm (d = −1.0 to −0.5 [−1.70, 0.29]), plus at 12 pm to 5 pm (d = −0.50 [−1.21, 0.29]). For the non-dominant hand, effect sizes were found in winter at 6 am to 12 pm (d = −0.5 [−1.21, 0.29]) and 12 pm to 5 pm (d = 0.50 [−0.29, 1.21]).

### Perceived fatigue

**Figure 2.**
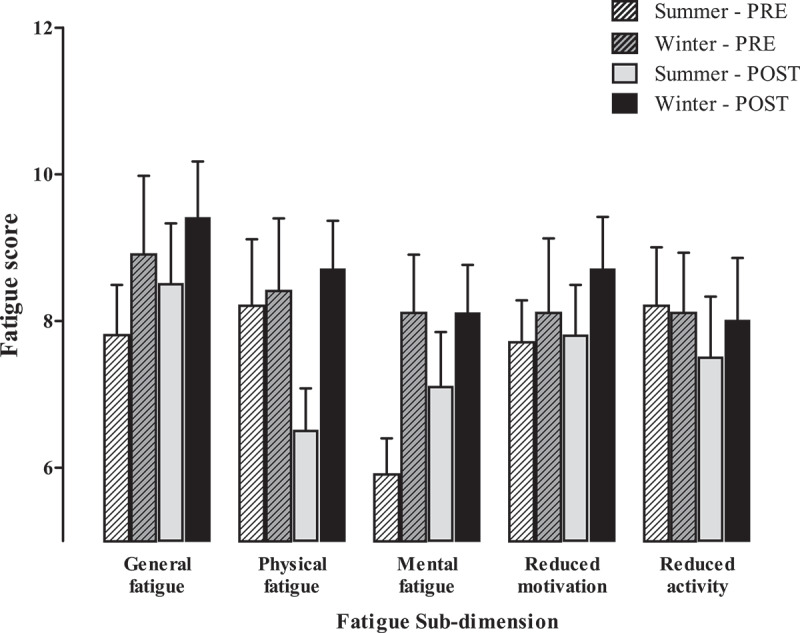
Sub-dimensions of multi-dimensional fatigue scale pre- and post-shift over summer (*n* = 13) and winter (*n* = 14).

No significant interaction effects were found for any fatigue sub-dimensions (*p* > 0.05) nor were there any main effects between seasons (*p* = 0.263) or for shift (*p* = 0.075). A large effect size for physical fatigue showed there was a tendency for fatigue at 5 pm in winter to be greater than summer (d = 1 [0.13, 1.72]). For motivation, a large effect size showed that in summer there was a tendency to have more motivation at 6 am compared to in winter at 6 am (Figure 2) (d = −0.78 [−1.50, −0.1]) .

### Physiological and perceptual variables

#### Core temperature

**Figure 3.**
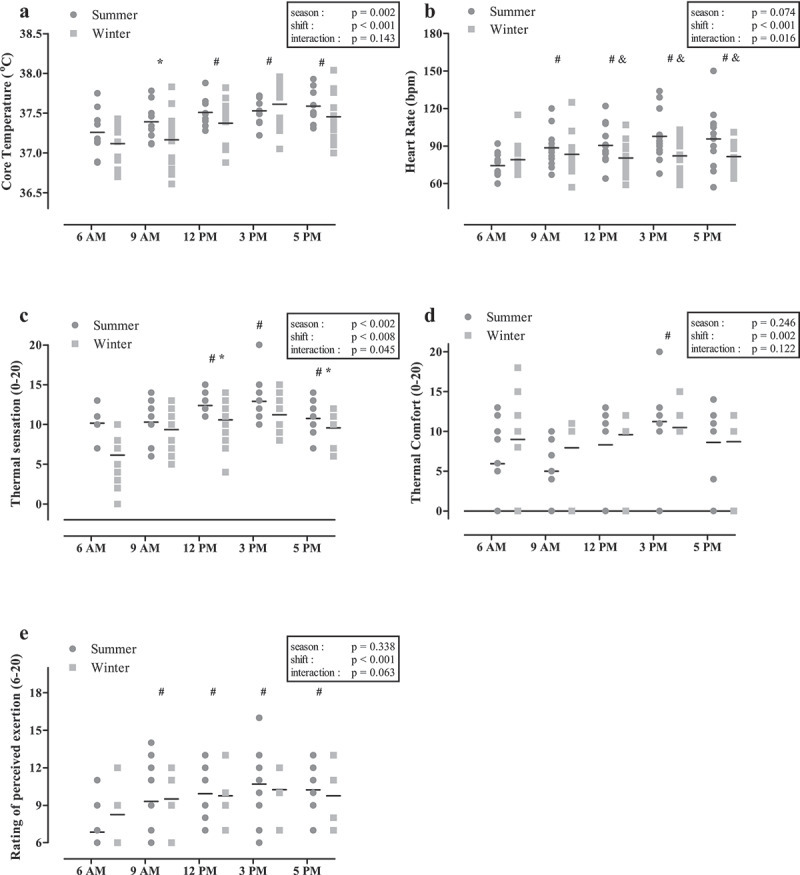
Core temperature (a); heart-rate (b); thermal sensation (c); thermal comfort (d); and ratings of perceived exertion (e) over the course of a shift in summer and winter seasons.

There was a main effect for season that demonstrated a significantly higher T_c_ in summer (37.46 ± 0.25°C; *n* = 9) compared to winter (37.34 ± 0.35°C, *p* = 0.002; *n* = 13). A main effect for shift demonstrated that T_c_ increased significantly by+0.33 ± 0.34°C (*p* < 0.001) over the course of the day. Compared to 6 am (37.18 ± 0.28°C), T_c_ was globally higher at 12 pm (37.43 ± 0.25°C; *p* < 0.001), 3 pm (37.58 ± 0.24°C; *p* < 0.001) and 5 pm (37.51 ± 0.29°C; *p* < 0.001). There was no significant interaction effect between season and shift for T_c_ (*p* = 0.143). Peak T_c_ in summer (37.59°C) occurred at 5pm, and in winter (Figure 3) (37.61°C) at 3pm.

#, different from 6 am, irrespective of season (p < 0.05)

*, different from previous time point, irrespective of season (p < 0.05) &;, different from 6 am in summer only (p < 0.05).

#### Heart rate

Mean heart-rate in summer (89 ± 18 bpm, *n* = 13) was not significantly different than in winter (81 ± 14 bpm, *n* = 14; *p* = 0.074). There was a significant main effect for shift (*p* < 0.001), with significantly higher heart-rate values at all time points when compared to 6 am. There was a significant interaction effect between season and shift for heart-rate (*p* = 0.016). Post hoc analysis revealed significantly lower heart-rate in summer at 6 am (74 ± 10 bpm) compared to 12 pm (90 ± 15 bpm; *p* = 0.029), 3 pm (98 ± 19 bpm; *p* < 0.001) and 5 pm (96 ± 23 bpm; *p* = 0.006).

#### Thermal sensation

Mean thermal sensation in summer (11 ± 2) was significantly greater than in winter (9 ± 3; *p* < 0.05). There was a significant interaction effect between season and shift for thermal sensation (*p* = 0.045), as well as a significant main effect for shift (*p* < 0.05). Thermal sensation in summer was significantly greater at 3 pm compared to 6 am (*p* = 0.019) and 9 am (*p* = 0.035). In winter, thermal sensation at 6 am was significantly lower than all other time points (*p* < 0.016). Thermal sensation was also greater at 6 am in summer than in winter (*p* < 0.001).

#### Ratings of perceived exertion

Mean RPE in summer (9 ± 3) did not differ significantly from winter (9 ± 2; *p* = 0.312). The main effect for shift was significant (*p* < 0.001) where, irrespective of season, RPE values were significantly greater at all time points of the shift when compared to 6 am (*p* ≤ 0.001). There was no significant interaction effect between season and shift (*p* = 0.063).

#### Thermal comfort

Thermal comfort did not differ significantly (*p* = 0.246) between summer (8 ± 5) and winter (9 ± 4). A main effect for shift was observed (*p* = 0.002) with scores for thermal comfort increasing from 6 am (7 ± 6) and 9 am (7 ± 5) to 3 pm (11 ± 3) indicating greater discomfort. There was no significant interaction effect for thermal comfort (*p* = 0.123).

#### Urinary specific gravity

**Figure 4.**
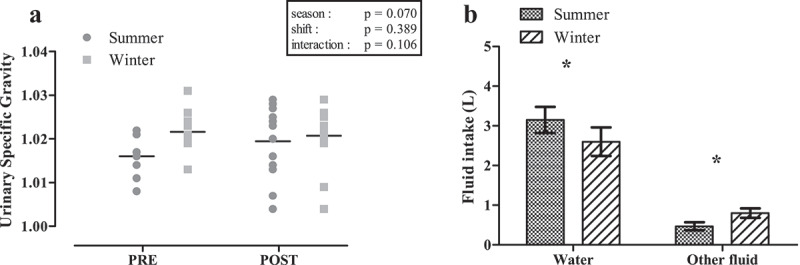
Urinary specific gravity changes from pre to post shift (a) and mean fluid intake over the course of a shift (b) in summer (*n*=13) and winter (*n*=14).

There was no significant interaction effect for USG (p = 0.106), nor were they any main effects for season (p = 0.070) or shift (p = 0.389; Figure 4a). Mean pre-shift USG in winter (1.022 ± 0.004) categorized participants as *“significantly dehydrated”* and in summer (1.016 ± 0.004) as *“minimally dehydrated”*. Post-shift values characterized participants as *“significantly dehydrated”* in winter (1.021 ± 0.007) and *“minimally dehydrated”* in summer (1.020 ± 0.008).

*, significantly different between summer and winter (p < 0.05).

#### Fluid intake

Total fluid intake during shift was significantly greater in summer than in winter (3.6 ± 1.1 *vs*. 3.3 ± 1.2 L; *p* = 0.025). Water intake in summer was significantly greater than in winter (3.2 ± 1.1 *vs*. 2.6 ± 1.3 L; *p* = 0.025; Figure 4b). There was a significant difference in other fluids consumed between seasons (summer: 0.5 ± 0.3 L; winter: 0.8 ± 0.4 L; *p* = 0.05).

#### Activity

There was no significant difference (*p* = 0.710) between the number of steps recorded in summer (7418 ± 2388) and winter (7725 ± 3311).

## Discussion

This study assessed psycho-physiological, manual dexterity and cognitive responses in FIFO workers during hot (summer) *versus* temperate (winter) environmental conditions over the course of an 11-h shift at the start of a 14-day swing. The main findings were that in summer compared to winter: (i) working memory scores were lower, (ii) perceived fatigue and manual dexterity performance did not differ, and (iii) mean T_c_ and thermal sensation were higher. While there were no significant changes in USG values, clinical descriptors [22] demonstrated that 23% of workers started work significantly dehydrated in summer with 54% concluding work significantly dehydrated. In winter, 64% started work significantly to seriously dehydrated and 64% finished work significantly dehydrated [24–28].

Exposure to summer heat resulted in globally lower (impaired) counting span scores and correct counting responses compared to winter. These results are consistent with previous literature that reported impaired complex cognitive task performance associated with increases in T_c_ due to heat exposure [29] and accompanying dehydration [30]. Notably, mean T_c_ in the current study did not reach the same peak levels as those reported by Gaoua et al. [29] (38.60 ± 0.1°C *vs* 37.46 ± 0.25°C), while Jimenez-Pavon et al. [30] only reported losses in body-mass as opposed to USG values, making direct comparison difficult. The difference in counting span score between seasons should however be interpreted with caution as only three participants were the same between seasons, hence the difference between performance may be due to participant differences. This promotes the possibility that changes in performance may at least partially be due to the fact that many of our participants were different people in summer compared to winter. Further, counting span scores did not differ over the course of the work shift, irrespective of season. This finding was unexpected, as previous research has reported increased errors, increased task duration and extended reaction time in cognitive tasks assessed in the heat from pre- to post-shift [6]. One potential explanation could be the lower-than-expected increase in T_c_ in the current study over the work shift, however as Mazloumi et al. [6] did not measure T_c_ this conjecture cannot be confirmed. In addition, while recall latency was not influenced by heat exposure, counting latency performance was slower at the beginning compared to the middle and end of a shift. It is well known that T_c_ rises throughout the day due to circadian rhythm, typically increasing by~0.5°C from early morning (4 am) to late afternoon (4–6 pm) [31]. Counting latency in our study was faster in the afternoon compared to the morning, which was similar to Craig et al. [32] where speed and ability to perceive stimuli were faster in the afternoon compared to the morning due to the effects of the circadian rhythm.

Performance on the manual dexterity task followed a similar pattern to recall and counting latency, whereby performance improved over the course of a shift but was not different between seasons. We hypothesized that performance would be hindered in summer compared to winter. This may not have occurred due to the smaller than expected increase in T_c_. The improved scores over the course of the shift may also be attributed to changes in circadian rhythm, which may have resulted in improved alertness and attention. Valdez [33] reported that, due to changes in circadian rhythm, attention which is lowest between 7 am to 10 am, improves between 10 am and 2 pm before dipping early afternoon, then increasing again early to late evening (4 pm to 8 pm). These timings coincide with when we assessed our participants and reflect the variations in results. Although the first testing session occurred after a short meeting, participants may still have been feeling tired or lethargic from the early morning start. This may have led to diminished alertness in the morning compared to after a day of active work [33].

Dehydration can result in an increased T_c_, poor concentration and the risk of developing heat related illnesses [34,35]. In our study, results for summer indicated that 9/13 (69%) workers finished their shift with a higher USG compared to pre-shift values, demonstrating increasing dehydration levels occurring over the course of the day. In winter, 8/14 (57%) of workers had a higher USG at the conclusion of their shift in winter. Our findings were consistent with previous research by Polkinghorne et al. [36] that reported pre-shift hydration status likely influences the outcome of hydration status at the end of a work shift, where 59% of underground miners started work with a USG>1.020 (significantly to seriously dehydrated), with 58% ending their shift with a value>1.020. This places workers at an increased risk of experiencing heat-related injury or illness that is often associated with dehydration in the workplace [37]. Dehydration has also been linked to impairment in cognitive performance, which may result in workplace injuries due to deteriorated concentration [37]. Nonetheless, no significant association was observed between dehydration and cognitive performance, as evidenced by weak correlations between USG values and cognitive parameters, despite dehydration being more pronounced in winter compared to summer in the current study. Furthermore, a greater decrement was found in cognitive performance in summer when hydration levels were better. Dehydration levels reported for workers in both seasons suggest that fluid intake over the course of the day was inadequate. Pre-shift dehydration levels in winter may have been higher when compared to summer values due to the lack of stimulus to consume fluids (i.e. lower thermal sensation scores at the commencement of the shift) and/or the possible reduced emphasis to drink in cooler conditions [38].

Perceived fatigue in workers did not differ between seasons or from pre- to post-shift. Comparing fatigue scores to studies in the mining industry and general population is anecdotal as most have assessed the accumulation of fatigue over the course of a swing (ranging from 7 to 28 days) including the changeover from day shift to night shift [39,40], which did not occur in the current study. Further studies that include an objective measure of fatigue or attention capacity (psychomotor vigilance tasks) and a measure of perceived fatigue and sleep may provide further insight into the consequences of fatigue on cognitive performance [41,42]. Regardless, working an 11-h shift in hot compared to temperate conditions did not result in greater sensations of fatigue in mine service workers.

Although our participants experienced a significant elevation in T_c_ over the course of the 11-hr shift (0.33 ± 0.34°C) during the summer months, while mean T_c_ in summer (37.46°C) was significantly higher than in winter (37.34°C), this elevation could be attributed to oscillations in T_c_ due to the circadian rhythm. Similar to findings by Girard et al. [7] and Peiffer et al. [2], average T_c_ values recorded in our study were below those generally associated with negative side effects of occupational heat stress and the onset of hyperthermia (T_c_>38°C [43,44]. Interestingly, heart-rate, thermal comfort and RPE did not differ between seasons despite a higher T_c_ in summer. This outcome may be a result of T_c_ not exceeding 38°C, thus minimizing an autonomic response designed to cool the body (i.e. increased sweating, redistribution of blood to the skin for cooling, and a consequent increase in heart-rate [35]), suggesting that workers were able to thermoregulate effectively in summer temperatures. Further, the higher fluid intake recorded in summer, compared to winter, would have assisted in improving hydration status [45] in workers as reflected by lower USG values, which in turn would have aided thermoregulation [46]. The maintenance of T_c_ may also have been due to the ability of the workers to self-pace [47]. Although there was no difference in activity/steps determined in summer compared to winter, upper body work was not assessed and this may have resulted in different metabolic loads associated with self-pacing based on season. It has been noted that workers with the ability to self-pace under thermally stressful conditions were able to mitigate physiological strain [48,49].

There were a number of limitations to this study. Due to the limited number of workers on site, our cohort consisted of a range of occupations that involved varying complexities and time spent on each job (i.e. not strictly quantified), as well as being limited to only males as there were no females in the current workforce. Therefore, it was not possible to measure productivity as work tasks can differ between and even within a specific occupation. Secondly, we did not record alcohol consumption on the night prior to their shift. The amount of alcohol consumed the night prior to work may have contributed to the dehydration status reported for workers prior to their shift commencing. This study only assessed workers on a single day at the start of a swing, future research should aim to assess workers on multiple days over the course of a swing to evaluate the effects of working on consecutive days in these environments. Future studies should include the assessment of skin as a driver of human performance/productivity. Specifically, skin temperature acts as a feedforward signal for behavioral thermoregulation (e.g. self-pacing), which can result in decreases in metabolic heat production associated with lower muscle activation [50]. Lastly, due to attrition rates over time, not all participants were assessed in both seasons, with only three individuals assessed in both winter and summer. This could be one factor that may have accounted for differences in cognitive performance and/or T_c_ variability between seasons, although multiple factors such as differences in aerobic fitness levels, acclimatization, or circadian rhythm may have contributed to differences. Again, this is reflective of a field-based study undertaken over the course of nearly a year.

## Conclusion

This was the first field-based mining study that assessed cognitive performance, manual dexterity and psycho-physiological responses in FIFO workers at the commencement of their swing in summer and winter. Working memory performance was impaired in summer compared to winter, with a high percentage of workers minimally to significantly dehydrated prior to and at the conclusion of their daily shift during both seasons. The effects of hot ambient conditions on dehydration are important considerations in respect to workers’ cognitive function, health (increased chance of kidney disease) and workplace safety. This is particularly of issue with environmental temperatures increasing due to climate change and the existence of heat waves. Workers need to remain vigilant to the symptoms of heat stress and partake in a work protocol that allows them to preserve their physiological and cognitive health, especially in the summer months.
